# Gut phageome of the giant panda (*Ailuropoda melanoleuca*) reveals greater diversity than relative species

**DOI:** 10.1128/msystems.00161-23

**Published:** 2023-06-05

**Authors:** Juan Lu, Haoning Wang, Chunmei Wang, Min Zhao, Rong Hou, Quan Shen, Shixing Yang, Likai Ji, Yuwei Liu, Xiaochun Wang, Songrui Liu, Tongling Shan, Wen Zhang

**Affiliations:** ^1^ Department of Laboratory Medicine, School of Medicine, Jiangsu University, Zhenjiang, Jiangsu, China; 2 School of Geography and Tourism, Harbin University, Harbin, Heilongjiang, China; 3 Shanghai Veterinary Research Institute, Chinese Academy of Agricultural Sciences, Shanghai, China; 4 Chengdu Research Base of Giant Panda Breeding, Sichuan Key Laboratory of Conservation Biology for Endangered Wildlife, Sichuan Academy of Giant Panda, Chengdu, Sichuan, China; Duke University School of Medicine, Durham, North Carolina, USA

**Keywords:** viral metagenomics, gut phageome, diversity, giant pandas, bacteriophages

## Abstract

The gut flora is a treasure house of diverse bacteriophages maintaining a harmonious and coexistent relationship with their hosts. The giant panda (*Ailuropoda melanoleuca*), as a vulnerable endemic species in China, has existed for millions of years and is regarded as a flagship species for biodiversity conservation. And yet, limited studies have analyzed the phage communities in the gut of giant pandas. Using viral metagenomic analysis, the phageomes of giant pandas and other relative species were investigated. Our study explored and compared the composition of phage communities from different animal sources. Giant pandas possessed more diverse and abundant phage communities in the gut compared with other relevant animals. Phylogenetic analyses based on the phage terminase large subunit (TerL) showed that the *Caudovirales* phages in giant pandas also presented highly genetic diversity. Our study revealed the diversity of phage communities in giant pandas and other relative species, contributing to the health maintenance of giant pandas and laying the groundwork for molecular evolution research of bacteriophages in mammals.

**IMPORTANCE:**

Gut phageome plays an important role in shaping gut microbiomes by direct interactions with bacteria or indirect influences on the host immune system, potentially regulating host health and disease status. The giant panda (*Ailuropoda melanoleuca*) is a vulnerable and umbrella species for biodiversity conservation. Our work explored and compared the gut phageome of giant pandas and relative species, contributing to the health maintenance of giant pandas.

## OBSERVATION

Bacteriophages, as the largest part of the gut virome, play pivotal roles in shaping gut microbiomes by direct interactions with their bacterial hosts or indirect influences on the host immune system (1, 2). A healthy gut generally has a phage–bacteria–host triangular relationship, with fruitful interactions and comparatively balanced structure (3). Bacteriophages that reside in the gastrointestinal tract are characterized by extraordinary stability and high diversity (4, 5). Although gut environments provide a bountiful source of bacteriophage genetic diversity, phages were more unexplored compared with bacteria and eukaryotic viruses (6). Megataxonomy of the virus world was actualized through a uniform standard based on viral hallmark genes (VHGs) that were widely conserved among various groups of viruses and provided a window to explore viral evolutionary relationships (7). Thereafter, the proposal was approved by the International Committee on the Taxonomy of Viruses. Tailed dsDNA phages, for instance, constitute the order *Caudovirales*, which possess conserved portal proteins and terminase subunits, and the terminase large subunits were generally considered as the VHGs of the order *Caudovirales* used for the phylogenetic analysis (7).

The giant panda (*Ailuropoda melanoleuca*) is a vulnerable indigenous species in China, and an umbrella species for biodiversity conservation (8). And the giant panda is considered as one of the oldest extant species with a reputation of “living fossils,” dating back to 8 million years ago (8). Owing to the joint efforts from every aspect of society, the protection level of the giant panda was degraded from “endangered” to “vulnerable” in the International Union for Conservation of Nature Red List of threatened species in 2016 (9). However, viral infectious diseases represent a serious health hazard to the giant panda. Several viral pathogens were considered to be a health hazard to giant pandas, such as the canine distemper virus (10), the canine parvovirus (11), and the feline panleukopenia virus (12), bringing the risk of cross-species infection. Nevertheless, bacteriophages, as the most abundant component of the gut virome, still remain to be explored in giant pandas. Therefore, in the present study, we explored the gut phageome of giant pandas and relevant species, and analyzed the genetic diversity of the *Caudovirales* order based on the terminase large subunits genes through phylogenetic analyses. These findings could reveal the potential interspecific diversity of gut bacteriophages among giant pandas and relative species, contributing to the health maintenance of giant pandas and providing a foundation for research into bacteriophages in the future.

To explore the gut phageome of giant pandas and other relevant animals, including bears, red pandas, and musk deer, we conducted viral metagenomics research of bacteriophages in 413 feces samples from giant pandas, 161 from red pandas, 70 from bears, and 85 from musk deer. These samples were pooled into 65 libraries according to animal sources and sample size. After next generation sequencing, the 65 libraries generated a total of 175,261,692 raw sequence reads with an average length of 247 bp and an average proportion of guanine (G) and cytosine (C) in the sequences (GC content [GC%]) of 45.8%. The sequence reads were binned according to barcode and were assembled into larger contigs. In total, 690,673 phage contigs were obtained through *de novo* assembly within the 65 libraries and alignment against the phage protein database using BLASTx (Table S1).

**FIG 1:**
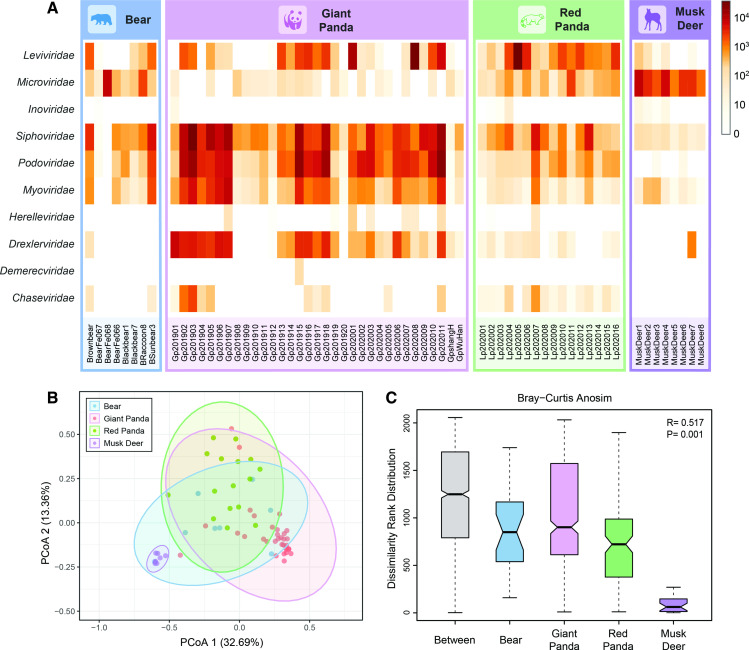
Comparison of gut phage communities of giant panda and other relevant animals. (**A**) Distribution heatmap of gut phage communities. Heatmap representing the reads number of each phage family in exponential form. The names of phage families are presented on the left, and the library names are presented at the bottom. Different animal sources are represented by rectangles with different colors and animal names are indicated on top; (**B**) principal coordinate analysis plot; (**C**) Analysis of similarities was performed among four animal groups. Different animal groups are marked with corresponding colors (see color legend).

To investigate the constitution and distribution pattern of gut phage communities in different animal groups, a series of comparative analyses were performed. The distribution heatmap of phage communities presented that the phage contigs and singlets reads of giant pandas, red pandas, bears, and musk deer were classified into 10 phage families (Fig. 1A). Thereinto, the phage communities of giant pandas were mainly dominated by *Siphoviridae*, *Podoviridae*, *Myoviridae*, and *Drexlerviridae* families, whereas the *Leviviridae* family was distributed sporadically. Unlike giant pandas, the most abundant phage family of red pandas is *Leviviridae*, and relatively low numbers of reads were classified into *Podoviridae*, *Myoviridae*, and *Drexlerviridae*. In addition, the family *Microviridae* accounted for the largest percentage of viral reads in the libraries of musk deer, whereas the phage communities of giant pandas possess the least abundant reads in the *Microviridae* family. Meanwhile, comparison of phage communities among giant pandas and other relevant animal groups through Bray-Curtis analysis of similarities (ANOSIM) and principal coordinate analysis (PCoA) suggested that the difference among groups was statistically significant with *P* < 0.01 (Fig. 1B and C). Furthermore, the greatest within-group difference was observed in the group of giant pandas, and the least difference was observed in musk deer (*P* < 0.05) (Fig. S1).

**FIG 2:**
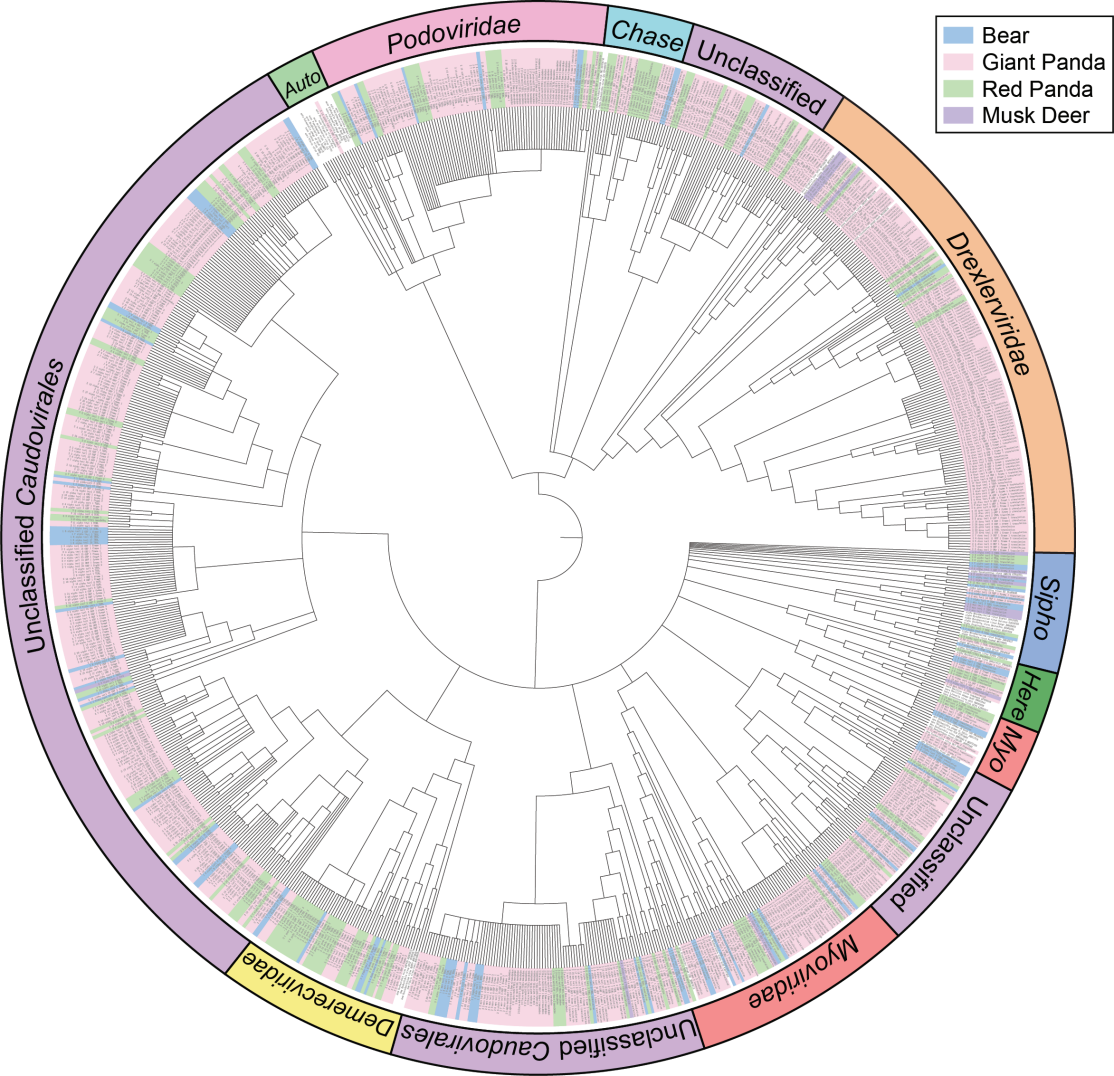
The phylogeny of *Caudovirales* identified in feces of giant panda and other relevant animals. Bayesian inference tree was established based on amino acid sequences of TerL of *Caudovirales*. Representative strains of all families in *Caudovirales* were included. The sequences of TerL found in this study are indicated in different colors according to animal groups (see color legend). Viral groups are marked around the tree with different colors. *Auto*, *Autographiviridae; Chase*, *Chaseviridae; Sipho*, *Siphoviridae; Myo*, *Myoviridae; Here*, *Herelleviridae*.

The order *Caudovirales* represents the largest category of bacteriophages, and the TerL region has high conservativeness of evolution in the *Caudovirales* order. To phylogenetically analyze phage sequences in the libraries of giant pandas and other animals, 944 sequences with complete coding sequence of phage terminase large subunits (TerL) were obtained after contigs annotation, including 87 sequences from the libraries of bears, 672 from giant pandas, 173 from red pandas, and 12 from musk deer (Table S2). The BLASTx results showed that these phage sequences shared sequence identities with their best matches ranging from 35.88% to 100%, and the length of them ranges from 1,224 bp to 3,090 bp with an average of 1,490 bp. The phylogenetic tree of bacteriophages was constructed based on the 944 TerL protein sequences identified in the study (Fig. 2). The topology structure of the phylogenetic tree revealed that a preponderance of TerL sequences did not cluster with any known families within the *Caudovirales* order, and formed four unclassified novel clusters among the known clades. Besides, several TerL sequences were phylogenetically clustered with known phage strains in the clades of *Drexlerviridae*, *Podoviridae*, *Myoviridae*, *Siphoviridae*, *Demerecviridae*, and so on. The cluster of the *Drexlerviridae* family is the largest of them, which was dominated by the sequences from giant pandas. Meanwhile, the clade of the *Demerecviridae* family is composed mainly of sequences from red pandas. And the sequences from musk deer were mainly clustered with bacteriophages in the *Siphoviridae* family. These results revealed the taxonomic composition and the potential diversity of bacteriophages in giant pandas and other relevant animals.

The order *Caudovirales* was the most essential part of bacteriophages and was ubiquitous not only in the natural environment but in the gut virome of human, which could indirectly interact with the host immune system to regulate health and disease (2, 5, 13). In the present study, the diversity and abundance of phage communities in the gut of giant pandas was significantly higher than other relevant animals, which was dominated by the order *Caudovirales*, mainly including *Siphoviridae*, *Podoviridae*, *Myoviridae*, etc. Previous research observed a correlation between the abundance of the order *Caudovirales* and inflammatory bowel disease. Thus, the effects of bacteriophages on intestinal health of giant pandas remain to be studied further (14). Meanwhile, bacteriophages are able to modulate the composition and abundance of gut bacteria, which could also be used for antibiotic therapy to treat bacterial infections (15). In the study, bacteriophages in the *Drexlerviridae* family of giant pandas were more abundant than that of other relevant animals. And a series of members of the *Drexlerviridae* family have been recognized as candidate phages in the development of phage therapy (16). Furthermore, phage terminase large subunit (TerL) gene is one of the virus hallmark genes and is broadly conserved in the order *Caudovirales* (7). The present study also revealed the hidden diversity of bacteriophages in giant pandas through phylogenetic analysis of the TerL gene, consistent with the research on human gut virome (17). Accordingly, although phages were typified by simple structure and tiny size, they owned the greatest abundance and the highest diversity in the gut virome. In addition, our study revealed a certain connection between the composition of phage communities and animal species. Because of the rarity of these animal species and the difficulty of sampling, animal sources and sample sizes are unavoidably limited. Besides, the gut phage communities are also subject to living environment and eating patterns (18). In this study, the timescale of sampling is over 2 yr; the change of diet in different seasons might influence the composition of gut microbiome. Previous research also revealed that environmental factors might play a more significant role in shaping gut microbiota than host genetics (19). Thus, further research would be needed to reveal the potential relationship in a broader and deeper vision. Ultimately, our research reveals the gut phageome of giant pandas and other mammals, providing a foundation for the giant panda protection efforts in the future and contributing a better understanding of the diversity and evolution of bacteriophages in mammals.

## METHODS

### Sample collection and preparation

During 2018 to 2020, to investigate the phageome of giant pandas and other relevant animals (including bears, red pandas, and musk deer), in total, 729 fecal samples were collected from Sichuan, Wuhan, Chongqing, and Shanghai in China using disposable materials (Table S1). Samples were pooled into 65 sample pools according to sample size and source, including 8 pools for bears, 33 for giant pandas, 16 for red pandas, and 8 for musk deer. Samples were re-suspended in 500 µL Dulbecco’s phosphate-buffered saline and vigorously vortexed for 5 min, following frozen and thawed three times on dry ice. The supernatants were then collected after centrifugation (10 min, 15,000 g) and stored at –80°C until use.

### Viral metagenomic library construction

Supernatant from each sample was pipetted and equally pooled into different sample pools to obtain the final volume of 500 µL. Sample pools were centrifuged at 12,000 g for 5 min at 4°C and then filtered through a 0.45 µm filter (Millipore). The filtrates enriched in viral particles were treated with DNase and RNase to digest unprotected nucleic acid (20 - 22). Then, the remaining total nucleic acid was isolated using QIAamp MinElute Virus Spin Kit (Qiagen) according to the manufacturer’s protocol. The viral nucleic acid samples were subjected to reverse transcription reactions using reverse transcriptase (Super-Script IV, Invitrogen) and 100 µmol of random hexamer primers, followed by a single round of DNA synthesis using Klenow fragment polymerase (New England BioLabs). Overall, 65 libraries were constructed using Nextera XT DNA Sample Preparation Kit (Illumina). All libraries were sequenced on an Illumina NovaSeq 6000 platform (23).

### Bioinformatics analysis

Paired-end reads generated by NovaSeq were debarcoded using vendor software from Illumina. An in-house analysis pipeline running on a 32 nodes Linux cluster was utilized to process the data. Reads were considered duplicates if bases 5–55 were identical and only one random copy of duplicates was kept. Low sequencing quality tails were trimmed using Phred quality score 30 as the threshold. Adaptors were trimmed using the default parameters of VecScreen with specialized parameters designed for adapter removal. Bacterial reads were subtracted by mapping to the bacterial nucleotide sequences from the BLAST non-redundant (nr) database using Bowtie2 v2.2.4. The cleaned reads were *de novo* assembled by SOAPdenovo2 version r240 using Kmer size 63 with default settings (24). The assembled contigs, along with singlets, were then matched against a customized viral proteome database using BLASTx with an E-value cutoff of <10^−5^. The virus BLASTx (v.2.2.7) database was compiled using the National Center for Biotechnology Information (NCBI) virus reference proteome and viral proteins sequences from the NCBI nr database. Candidate viral hits are then compared to an in-house non-virus non-redundant (NVNR) protein database with an E-value cutoff of <10^−5^ to remove false-positive viral hits. The NVNR database was compiled using non-viral protein sequences extracted from the NCBI nr database. Contigs without significant BLASTx similarity to viral proteome database are searched against viral protein families in the vFam database (25) using HMMER3 (26 - 28) to detect remote viral protein similarities.

### Viral community analysis

Composition similarity analysis of the 65 viromes were compared using MEGAN software (v6.21.7) (29) under the compare option. The results were presented using the Unweighted Pair Group Method with PCoA under Bray-Curtis ecological distance matrix with default parameters. ANOSIM was used to compare differences among groups using R v4.0.4 package vegan (v2.5.7). The viral community structure and richness results were visualized in the heatmap which was generated using R v4.0.4 package pheatmap (v1.0.12).

### Viral sequences extension and annotation

Viral contigs were merged using the Low Sensitivity/Fastest parameter in software Geneious v11.1.2 (30). And the individual contig was used as reference for mapping to the raw reads of its original barcode using the Low Sensitivity/Fastest parameter. Putative viral open reading frames (ORFs) were predicted by Geneious v11.1.2 with built-in parameters (minimum size: 300; genetic code: Standard; start codons: ATG) (30) and were checked through BLASTp in NCBI. The annotations of these ORFs were based on comparisons to the Conserved Domain Database with an E-value cutoff of <10^−5^. Those contigs annotated with phage terminase large subunits (TerL) of *Caudovirales* were selected, among which identified as complete ORFs were included for further phylogenetic analyses.

### Phylogenetic analysis

Phylogenetic analysis was performed based on the protein sequences of phage terminase large subunits (TerL) identified in this study and protein sequences of reference strains belonging to different families of *Caudovirales*. These protein sequences were aligned using MUSCLE in MEGA v10.1.8 with the default settings (31). Sites containing more than 50% gaps were temporarily removed from alignments. Bayesian inference trees were then constructed using MrBayes v3.2.7 (32). The Markov chain was run for a maximum of 1 million generations, in which every 50 generations were sampled and the first 25% of Markov chain Monte Carlo samples were discarded as burn-in. Maximum Likelihood tree was also constructed to confirm the Bayesian inference tree using software MEGA v10.1.8 (31).

## Data Availability

The raw sequence read data analyzed in this study are available at the NCBI Sequence Read Archive database under the accession numbers listed in Table S1. All viral sequences of phage terminase large subunit (TerL) identified in this study were deposited in the GenBank database under the accession numbers from OP517077 to OP518020 (Table S2).

## SUPPLEMENTAL MATERIAL

Figure S1 msystems.00161-23-s0001.tif Analysis of similarity (ANOSIM) between groups based on the Bray-Curtis distances.

Table S1 msystems.00161-23-s0002.xlsx Information of sample source and corresponding libraries.

Table S2 msystems.00161-23-s0003.xlsx Information of viral sequences with phage terminase large subunit (TerL) identified in the present study.

ASM does not own the copyrights to Supplemental Material that may be linked to, or accessed through, an article. The authors have granted ASM a non-exclusive, world-wide license to publish the Supplemental Material files. Please contact the corresponding author directly for reuse.
