# Supplementary figures and images for: Gut phageome of the giant panda (Ailuropoda melanoleuca) reveals greater diversity than relative species
Source: mSystems. 2023 Jun 5;8(3):e00161-23. doi: 10.1128/msystems.00161-23 (PMC10308893; doi:10.1128/msystems.00161-23)

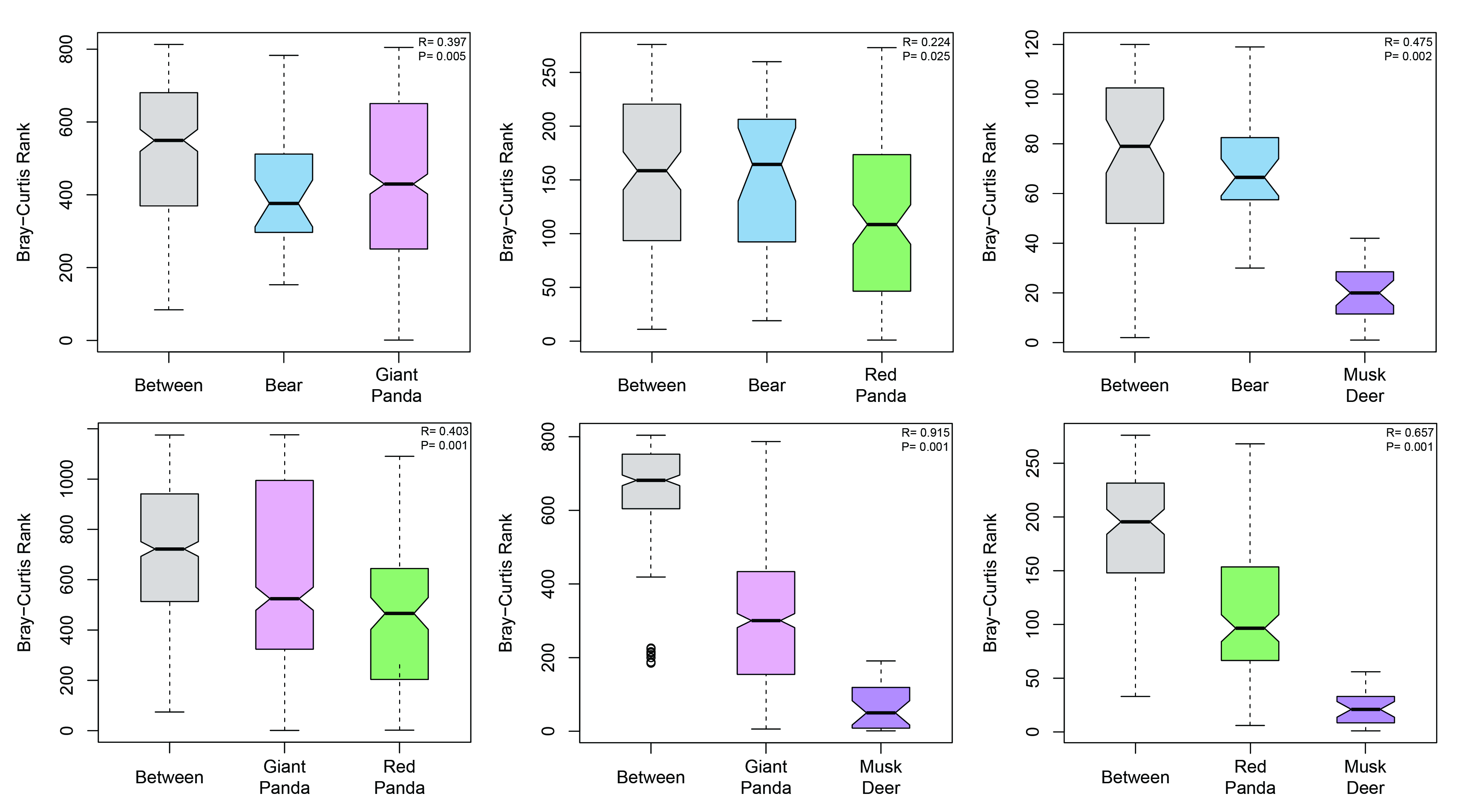

Supplement: Figure S1 — Analysis of similarity (ANOSIM) between groups based on the Bray-Curtis distances. [file msystems.00161-23-s0001.tif]
